# Supplementary material for: Isolation of a T7-Like Lytic Pasteurella Bacteriophage vB_PmuP_PHB01 and Its Potential Use in Therapy against Pasteurella multocida Infections
Source: Viruses. 2019 Jan 21;11(1):86. doi: 10.3390/v11010086 (PMC6356340; doi:10.3390/v11010086)
Supplement: Supplementary file 1 [file viruses-11-00086-s001.pdf]

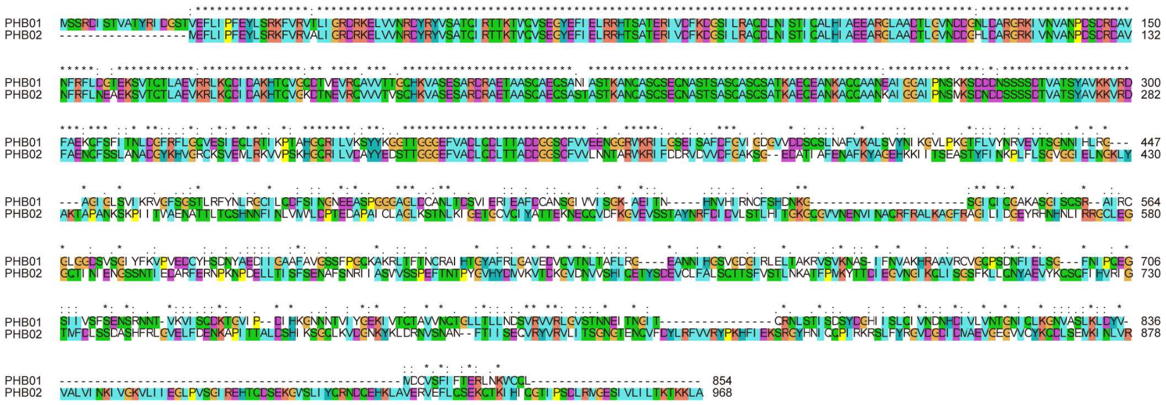

Figure S1. Sequence comparison of the tail fiber protein encoded by PHB01 and PHB02.

Table S1. The annotation of phage PHB01 genome.

ORFs	Start	Stop	Length(aa)	Function
1	647	790	47	
2	1103	1402	99	
3	1389	1673	94	
4	1730	1957	75	
5	1990	4593	867	T7 RNA polymerase
6	4606	4740	44	
7	5045	6043	332	DNA ligase [Enterobacter phage E-2]
8	6156	6521	121	hypothetical protein [Methylomonas]
9	6514	7035	173	hypothetical protein [Yersinia phage YpP-G]
10	7062	7178	38	
11	7175	7492	105	hypothetical protein [Salmonella virus SPC35]
12	7485	7673	62	
13	7696	8391	231	ss DNA binding protein [Yersinia phage Yep-phi]
14	8394	8846	150	endonuclease I [Enterobacteria phage K30]
15	8846	9298	150	endolysin [Morganella phage vB_MmoP_MP2]
16	9312	10985	557	DNA primase/helicase [Yersinia phage phiYeO3-12]
17	10985	11149	54	
18	11130	11429	99	
19	11426	11659	77	hypothetical protein [Klebsiella phage vB_Kp1]
20	11718	13868	716	T7-like phage DNA polymerase [Yersinia phage YpP-Y]
21	13878	14132	84	
22	14125	14334	69	phage HNS binding protein [Enterobacteria phage T7]
23	14331	14609	92	
24	14750	14983	77	hypothetical protein [Roseobacter phage DSS3P8]
25	14973	15878	301	exonuclease [Escherichia phage P694]
26	15923	16192	89	hypothetical protein [Faecalibacterium sp. CAG:82-related_59_9]
27	16192	16380	62	hypothetical protein [Yersinia phage YpP-G]
28	16380	16676	98	putative tail assembly protein [Pectobacterium phage PP81]
29	16679	18307	542	head to tail joining protein [Yersinia phage vB_YenP_AP10]

30	18322	19197	291	capsid assembly protein [Erwinia phage vB_EamP-L1]
31	19243	20250	335	major capsid and protein [Klebsiella phage vB_KpnP_IME205]
32	20322	20909	195	tail tubular protein A [Yersinia phage vB_YenP_AP10]
33	20915	23293	792	phage tail fiber protein [Stenotrophomonas phage IME15]
34	23316	23825	169	putative internal virion protein A [Pseudomonas phage PPpW-4]
35	23804	24340	178	phage internal (core) protein [Klebsiella phage vB_KpnP_KpV289]
36	24333	26549	738	internal virion protein C [Klebsiella phage KP32]
37	26563	30648	1361	internal virion protein D [Escherichia phage CICC 80001]
38	30702	33266	854	tail fibers protein [Escherichia phage 64795_ec1]
39	33407	33655	82	class II holin [Klebsiella phage vB_KpnP_IL33]
40	33642	33893	83	DNA packaging protein A [Yersinia phage Berlin]
41	33908	34375	155	endopeptidase [Erwinia phage vB_EamP-L1]
42	34372	34794	140	hypothethical protein [Gordonia desulfuricans]
43	34787	36556	589	phage DNA maturation protein [Stenotrophomonas phage IME15]
